# CRISPR-to-Kill (C2K)–Employing the Bacterial Immune System to Kill Cancer Cells

**DOI:** 10.3390/cancers13246306

**Published:** 2021-12-15

**Authors:** Dawid Głów, Cecile L. Maire, Lea Isabell Schwarze, Katrin Lamszus, Boris Fehse

**Affiliations:** 1 Research Department, Cell and Gene Therapy, Department of Stem Cell Transplantation, University Medical Center Hamburg-Eppendorf (UKE), 20246 Hamburg, Germany; d.glow@uke.de (D.G.); l.schwarze@uke.de (L.I.S.); 2 Department of Neurosurgery, University Medical Center Hamburg-Eppendorf (UKE), 20246 Hamburg, Germany; cmaire@uke.de (C.L.M.); lamszus@uke.uni-hamburg.de (K.L.)

**Keywords:** CRISPR/Cas9, cancer gene therapy, programmed cell death, suicide gene, SINE, Alu, irradiation, glioblastoma, resistance, LeGO vectors

## Abstract

**Simple Summary:**

Reasoning that multiple DNA breaks will trigger programmed cell death, we generated lentiviral CRISPR-to-kill (C2K) vectors targeting highly repetitive SINE sequences for cancer gene therapy. In proof-of-concept experiments, C2K-Alu-vectors selectively killed human, but not murine cell lines, and efficiently inhibited the growth of patient-derived glioblastoma cell lines resistant to high-dose irradiation. In combination with tumor-targeting approaches, the C2K system might represent a promising tool for cancer gene therapy.

**Abstract:**

CRISPR/Cas9 was described as a bacterial immune system that uses targeted introduction of DNA double-strand breaks (DSBs) to destroy invaders. We hypothesized that we can analogously employ CRISPR/Cas9 nucleases to kill cancer cells by inducing maximal numbers of DSBs in their genome and thus triggering programmed cell death. To do so, we generated CRISPR-to-kill (C2K) lentiviral particles targeting highly repetitive Short Interspersed Nuclear Element-Alu sequences. Our Alu-specific sgRNA has more than 15,000 perfectly matched target sites within the human genome. C2K-Alu-vectors selectively killed human, but not murine cell lines. More importantly, they efficiently inhibited the growth of cancer cells including patient-derived glioblastoma cell lines resistant to high-dose irradiation. Our data provide proof-of-concept for the potential of C2K as a novel treatment strategy overcoming common resistance mechanisms. In combination with tumor-targeting approaches, the C2K system might therefore represent a promising tool for cancer gene therapy.

## 1. Introduction

Suicide gene therapy (SGT) was proposed as a new therapy option for difficult-to-treat cancer more than 40 years ago (reviewed, e.g., in [1,2,3]). Current concepts in oncology mainly rely on the genetic modification of cancer cells to express prodrug-converting enzymes generating toxic metabolites in the presence of the respective prodrug, the most popular one being the Herpes simplex virus thymidine kinase (HSV-TK)/ganciclovir system. Several clinical studies provided promising results for SGT [4,5], but the use of prodrug-converting enzymes was also associated with inherent technical limitations. For HSV-TK, the latter included the presence of inactive splicing variants, cell-cycle dependence, slow killing kinetics and limited tissue penetration as well as myelotoxicity of the prodrug [6]. Clinical efficacy was also hampered by the pronounced treatment resistance of established tumors, particularly due to non-dividing (“dormant”) tumor stem (or tumor-initiating) cells [7]. Consequently, broad efforts were made to improve the current SGT systems [3,6,8,9]. However, in order to address tumor- and potentially recurrence-initiating cells [10], completely novel, cell-cycle independent SGT approaches with low probability for resistance development might be necessary.

Evolutionary, the CRISPR/Cas system represents a sophisticated prokaryotic adaptive “immune system” providing sequence-specific protection against foreign nucleic acids, particularly invading bacteriophages [11,12,13]. Even though CRISPR/Cas sequences were already discovered more than 30 years ago, only the elucidation of its mode of action in the early 2010s led to the development of a very broad range of applications for genome editing [14,15,16]. Today, genome editing commonly involves the introduction of single, targeted double-strand breaks (DSBs) to modify a chosen gene or locus. Using more than one guide-RNA, several loci can be targeted simultaneously [17], but such multiplexing has been associated with higher toxicity and genomic instability [18,19]. Indeed, the introduction of multiple DSBs is a common approach to kill cancer cells. For example, DNA-damaging mechanisms of irradiation underlie the wide use of radiotherapy in cancer treatment. Therefore, we hypothesized that the concurrent introduction of large numbers of DSBs by the CRISPR/Cas system should result in irreversible cell-cycle arrest and eventually programmed cell death (PCD)/apoptosis even in cells resistant to standard treatments.

The human as well as other eukaryotic genomes contain noncoding repetitive elements of several types. Some of them can be found thousands of times; we conjectured that these repetitive elements can be used to introduce large numbers of DSBs into the human genome with one single guide RNA (gRNA). Most likely, none of the existing molecular DNA repair mechanisms can overcome such cytotoxic stress; therefore, programmed cell death should be efficiently triggered. To test our assumption, we chose as target the Alu element–a 282-nt long Short Interspersed Nuclear Element (SINE) found approximatively 1.1 × 10^6^ times in the human genome [20]. We designed all-in-one lentiviral vectors encoding Cas9 in conjunction with an Alu-specific gRNA and a fluorescence marker; vector particles were used to transduce various human and murine cell lines. We here provide proof of principle that the new suicide system designated as CRISPR-to-kill (C2K) facilitates the efficient killing of a variety of human cells, including largely treatment-resistant patient-derived, “stem-like” glioblastoma cell lines [21,22,23]. Altogether, our results indicate that the C2K system can overcome inherent treatment resistance of highly aggressive cancer types or serve as co-treatment, e.g., to lower therapeutic irradiation doses.

## 2. Materials and Methods

### 2.1. Cell Lines and Cell Culture

The HEK-293T (ATCC CRL-3216), U87, G62, murine NIH-3T3, and their derivatives were cultured in Dulbecco’s modified Eagle’s medium (DMEM Glutamax, Thermo Scientific, Waltham, MA, USA) supplemented with 10% FCS, L-glutamine (2 mM), 100 U/mL penicillin, and 100 μg/mL streptomycin. For the Jurkat T cell line, instead of DMEM we used Roswell Park Memorial Institute 1640 medium (RPMI 1640, Thermo Scientific, Waltham, MA, USA).

Glioblastoma patient-derived “stem-like” cell lines GS-8 [21], NCH644 [22], and BT112 [23] were routinely cultured as neurospheres in serum-free Neurobasal Medium (NBM, Invitrogen, Carlsbad, CA, USA) supplemented with 1% Glutamine (Invitrogen), 2% B27 (Invitrogen), and 20 ng/mL of each epidermal growth factor and fibroblast growth factor–2 (PeproTech, Rocky Hill, NJ, USA).

Cell culture was performed under standard conditions (37 °C, 100% relative humidity, 5% CO_2_). Cell-culture material was purchased from Corning (Corning, NY, USA), Greiner Bio One (Frickenhausen, Germany), and Sarstedt (Nümbrecht, Germany).

### 2.2. Molecular Cloning

All-in-one CRISPR-Cas9 “LeGO-CC” vectors were generated by cloning the humanized *Streptococcus pyogenes* Cas9 (SpCas9) gene behind the internal SFFV promotor and chimeric guide RNA (gRNA) scaffold from pX330 (Addgene #42230), a kind gift from Feng Zhang [17], behind the U6 promotor of LeGO-iG2 (Addgene #27341) and LeGO-iC2 (Addgene #27345) vectors [24]. To generate constructs that express the gRNAs of choice, the respective sequences with the addition of ACC at the 5′ end of the leading strand, followed by G, if necessary (required for polymerase-II dependent transcription), and AAC at 5′ end of complementary strand were synthesized. ACC/AAC were added to allow for cloning pre-annealed F/R-Alu-gRNA oligos into the SapI cloning site of the LeGO-iC2-CC and/or LeGO-iG2-CC vectors. Primer sequences are provided in Table S1.

LeGO-eGFP-NLS-p53BPpuro+ vector was generated by cloning nls53BP1 from pcDNA-FRT/T0-eGFPnls-53BP1 1220-1631 WT (Addgene #60814) [25], a kind gift from Daniel Durocher, and 2Apuro from LeGO-iC2puro+ into the LeGO-G2 vector (Addgene #25917) [24] using the restriction site BsrGI. Primer sequences and cloning details are provided in Table S1.

### 2.3. Lentiviral Vector Production and Cell Transduction

VSV-G pseudotyped, third-generation all-in-one LeGO-CC vectors were produced in accordance with our standard protocols [24,26]. In short, 5 × 10^6^ cells were seeded and transfected with appropriate vector and packaging plasmids at established concentrations after overnight culture. Medium was exchanged after 6 h and the viral supernatant was harvested and filtered 24 h later. All-in-one LeGO-CC vector particles were concentrated by centrifugation (4 °C, 16 h, 8000× *g*).

For titration 10^5^ NIH-3T3 (obtained from ATCC (#CRL-1658)) cells/well were plated in 500 µL medium in a 24-well plate. Two hours later polybrene (8 µg/mL) and viral supernatants were added (in triplicates). After 24 h medium was exchanged to the standard growth medium. To estimate vector titers, cells were analyzed approximately 72 h after transduction. For other purposes, cells were analyzed at different time points as indicated in the manuscript.

Transduction of other cells was performed as indicated within the text.

### 2.4. Cytotoxicity Assay

48 to 72 h after transduction, 2 × 10^5^–10^6^ cells were washed with PBS, centrifuged, and resuspended in 100 µL Apoptosis Buffer (BioLegend, San Diego, CA, USA) Subsequently, Annexin V-APC conjugate (Clontech, Palo Alto, CA, USA) and PI solution were added to the cells. Samples were vortexed and incubated for 15 min at RT in the dark. Staining was stopped by the addition of 300 µL Apoptosis Buffer. Cells were analyzed by flow cytometry.

### 2.5. Cell-Cycle Analysis

A total of 10^6^ cells were washed with PBS and fixed with methyl alcohol. After incubation for at least 4 h at −20 °C, cells were washed twice with PBS and finally resuspended in PBS with 25 μg/mL PI solution (Thermo Scientific, Waltham, MA, USA) supplemented with 2.5 μg/mL RNase A (Thermo Scientific, Waltham, MA, USA). Cells were incubated for 30 min at 37 °C in the dark and analyzed by Flow Cytometry.

### 2.6. Flow Cytometry

Flow cytometry was performed on LSR Fortessa, FACS Canto II (both BD Biosciences, Ashland, OR, USA), NovoCyte Quanteon (Agilent, Santa Clara, CA, USA) or CytoFLEX (Beckman Coulter, Brea, CA, USA). Data were analyzed using BD FACSDiva and FlowJo (both BD Biosciences, Heidelberg, Germany).

### 2.7. Imaging Flow Cytometry

72 h after co-transduction with LeGO-iC2-C2K and LeGO-eGFP-NLS-p53BPpuro+, cells were harvested, washed with PBS, and resuspended in 200 µL PBS. Subsequently, Hoechst 33342 Nuclear Staining was added to the samples. After 5 min of incubation, cell images were obtained using the ImageStreamX Mk II System (Amnis/Luminex, Austin, TX, USA); data were acquired and analyzed with the IDEAS Software package (Amnis). GFP- (Tp53BP-GFP, Tp53BP-GFP + Zeocin) or GFP/mCherry- (Tp53BP-GFP + Cas9/Alu) positive cells were gated, and their images were investigated. Normal erode, Spot, and Peak image masques were successively applied to all images before counting GFP spots representing numbers of DSBs.

### 2.8. Colony Formation Assay

U87 cells were irradiated with the indicated doses and C2K was added 6 h after irradiation. Cell survival was determined by colony formation after delayed plating. After 24 h incubation cells were counted and 250 cells were seeded in 6-well plate format. After 3–4 weeks, the number of colonies containing more than 50 cells was assessed.

### 2.9. MTT Assay

Some 1 × 10^4^ or 5 × 10^4^ glioblastoma cells were plated in 100 μL medium per well of a 96-well plate and incubated at 37 °C (d0). After 24 h cells were irradiated, vector supernatant was added 6 h later. For analysis whole samples were moved to black, flat-bottom plates, and volume was adjusted to 100 μL, if necessary. A total of 100 μL of CellTiter-Glo Reagent (CellTiter-Glo Luminescent Cell Viability Assay, Promega, Madison, WI, USA) were added. MTT assay was performed following the manufacturer’s protocol.

### 2.10. X-ray Irradiation

All of the cells were irradiated in Gulmay RS225 X-ray source (Gulmay Medical Ltd., Surrey, UK) at room temperature with 200 kV X-rays (200 kV, 15 mA, 0.8 mmBe + 0.5 mm Cu filtering). By adjusting the distance between sample and X-ray source, the dosage was set to 1.2 Gy/min.

### 2.11. Statistical Analysis

Datasets shown as bar graphs with error bars show the average and standard deviation (SD) of three independent experiments, if not specified otherwise. Statistical significance was determined using a two-tailed, homoscedastic Student’s *t*-test. For all statistical analyses, the GraphPad Prism 7 software package was used (GraphPad Software, San Diego, CA, USA).

## 3. Results

### 3.1. Targeting Alu-SINE with CRISPR-Cas9 Leads to Strong Growth Inhibition of Human Cells

**Figure 1:**
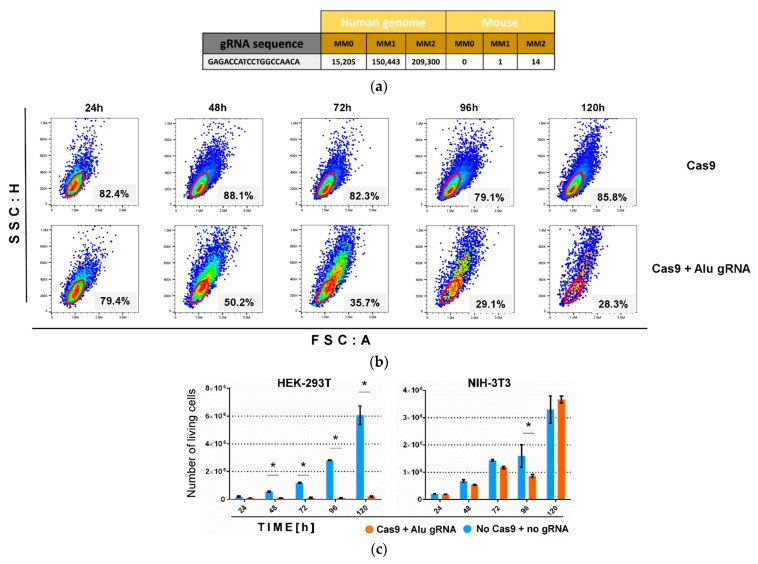
Targeting Alu SINE with Cas9 efficiently inhibits growth in human cells. (**a**) The sequence of Alu-gRNA used in the experiments. Numbers of sequences within human and mouse genomes identical (MM0), or containing one (MM1) or two (MM2) mismatches to Alu-gRNA. (**b**) Transduction with Cas9 and Alu-sgRNA causes strong and increasing-over-time changes in scatter characteristics of HEK293T cells as evident in the density plots based on the live-cell gate (red) with the contained cell proportions (in percent). In contrast, no major change in scatter characteristics was seen in the Cas9 only control (upper panel). (**c**) Growth inhibition of HEK-293T and NIH-3T3 cells after Alu-targeted C2K. Absolute numbers of living cells were counted by flow cytometry in defined volumes. Shown are cell numbers extrapolated to the volume of single wells. Data are presented as mean ± SEM, *n* = 3, * *p* < 0.05. See also Figure S2. In (**b**,**c**), time points of analyses are indicated (hours after transduction).

Reasoning that there should be a threshold of acceptable and repairable DSBs in any cell, we aimed to cause maximal damage by the introduced designer nuclease. Consequently, we chose as CRISPR/Cas target the 282-nt long Alu element (Figure S1A), of which >1 Mio highly homologous copies are present in the human genome. Exploiting the Cas-OFFinder of CRISPR RGEN [27] we identified an Alu-specific sgRNA with >15,000 perfectly matched target sites and additional >150,000/>209,000 (off-) targets with single and double mismatches (Figure 1a). In total >900,000 sequences containing ≤3 mismatches to the Alu-sgRNA were found in the human genome. In contrast, there was no perfect match of the chosen sgRNA to the mouse genome and only very low numbers of potential off-targets with 1 and 2 mismatches (Figure 1a). The Alu-sgRNA sequence was cloned into all-in-one LeGO-iG2-CC and LeGO-iC2-CC vectors; the resulting LeGO-iG2/iC2-Cas9/Alu-gRNA vectors were designated LeGO-iG2-C2K and LeGO-iC2-CC (Figure S1B).

To test the ability of C2K vectors to induce cell death, we first transduced human embryonic kidney (HEK-293T) cells and murine fibroblasts (NIH-3T3) with LeGO-iG2-C2K. We applied multiplicities of infection of approximately six expected to result in ~90% transduction efficiency [28]. Starting 24 h after transduction, GFP-positive human, but not murine cells showed phenotypic changes including altered scatter characteristics in flow cytometry, which became more pronounced over time (Figure 1b). These phenotypic changes were accompanied by strong growth inhibition reaching ≥95% 120 h post-transduction (Figure 1c). Importantly, no such effect was seen in murine fibroblasts (Figure 1c). To exclude any cell-specific effect, we next tested C2K on two further human cell lines-Jurkat T cells and G62 glioma cells. Again, we observed essentially complete suppression of cell growth (Figure S2). Together these data proved that targeting repetitive genomic loci by CRISPR/Cas could serve as a very efficient means to inhibit proliferation.

### 3.2. Alu-Directed C2K Causes Multiple DSBs Triggering Cell-Cycle Arrest and Apoptosis in Human Cells

According to our hypothesis, the growth inhibition observed in human cells was due to the induction of large numbers of DSBs not compatible with cell survival. To prove this assumption, we made use of an eGFP-coupled TP53-binding protein (TP53BP/GFP). TP53BP is involved in the early cellular response to DNA damage; the fusion protein was previously shown to facilitate the efficient marking of DSBs by GFP spots [29]. In order to co-express TP53BP/GFP and C2K components, we co-transduced HEK-293T cells with LeGO-eGFP-NLS-p53BP1 and LeGO-iC2-C2K vectors (Figure S1B).

**Figure 2:**
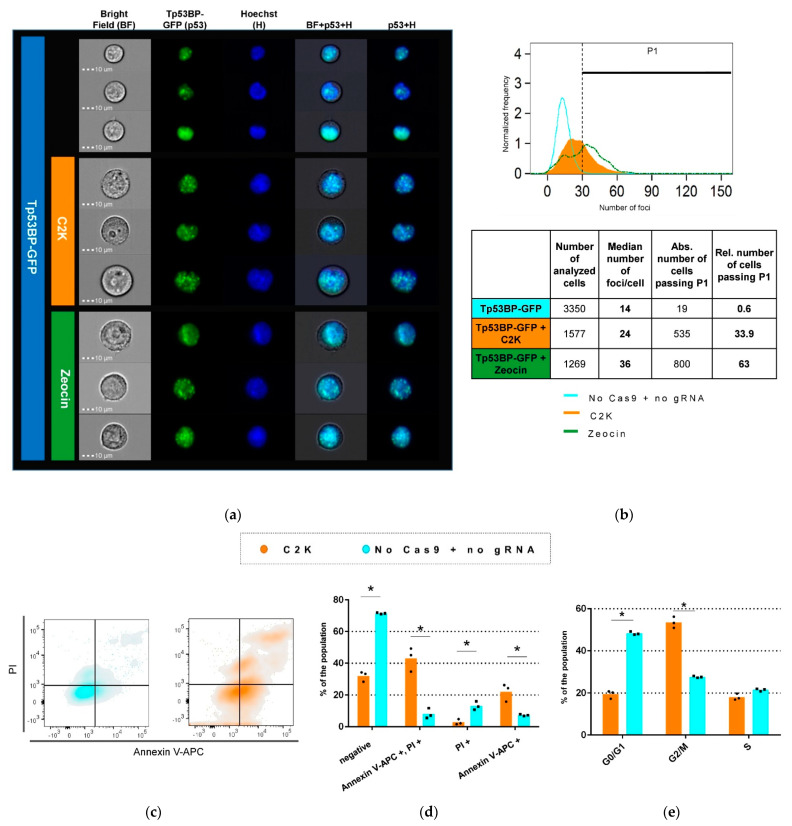
DSBs generated by Alu SINE-targeting Cas9 trigger cell-cycle arrest and apoptosis in human cells. (**a**) Visualization of DSB accumulation in cells treated with LeGO-iC2-C2K by imaging flow cytometry. Three representative images are shown per treatment group. Tp53BP-GFP positive cells treaded with Zeocin serve as the positive control. (**b**) DSBs quantification. Distribution of the eGFP foci count representing DSBs in the population of the cells treated with LeGo-iC2 –C2K or Zeocin. Numbers of eGFP foci representing DSBs were counted. P1 was set on 30 spots. (**c**,**d**) Apoptosis induction by C2K. LeGO-iC2-C2K-transduced and control cells were co-stained with Annexin V and propidium iodide (PI) and analyzed for the occurrence of early apoptotic (Annexin V+, gate 4) and late apoptotic/dead (Annexin V+/PI+, gate 2) cells. Individual Ddata points are (black boxes) and presented as mean values (orange and blue columns) are shown (*n* = 3) ± SEM. (**e**) G2/M cell cycle arrest caused by C2K. Cells were subjected to cell-cycle analysis by PI staining between 48 and 78 h after transduction. Percentages of cells in each phase of the cell cycle are shown. Data are presented as mean ± SEM, *n* = 3, * *p* < 0.05.

As in the previous series of experiments, 72 h post-transduction C2K cells displayed a markedly changed phenotype, as this time assessed by imaging flow cytometry (Figure 2a). Notably, the pictures seen in the bright field were very similar to those observed for cells treated with Zeocin, an antibiotic with well-described DNA-damaging activity. As compared to non-treated controls, numbers of DSB marked in individual cells by eGFP-TP53BP spots increased by factor 2 in C2K- and by a factor of 3 in Zeocin-treated cells-median numbers of eGFP spots were 12 for control, 24 for C2K-, and 36 for Zeocin-treated cells (Figure 2b). Given the different modes of action (immediate and continuous activity of the antibiotic versus delayed activity of C2K requiring integration and expression of the lentiviral vectors), the higher numbers of DSBs observed in the Zeocin group at this time point were not surprising. Notably, no increase in DSBs as compared to the control was observed in mouse cells treated with Alu-directed C2K vectors.To confirm that the observed growth inhibition was caused by C2K-induced DSBs leading to programmed cell death (PCD)/apoptosis, we next performed two flow-cytometry-based viability assays on LeGO-C2K-transduced cells. First, we measured apoptosis by 7-AAD/PI staining 72 h post-transduction (Figure 2c). A total of 65.1% of C2K-transduced cells were in the early or late stages of apoptosis, whereas the same was true for 15.4% of control cells, only (Figure 2d). Next, we applied PI staining to study the cell-cycle progression of C2K-transduced vs. control cells. As evident from Figure 2e, C2K-treated cells showed a clear G2/M arrest 72 h post-transduction (>50% of cells), consistent with the above described phenotypic changes.

### 3.3. C2K Efficiently Inhibits Growth, Triggers PCD, and Increases Radiosensitivity in Patient-Derived Glioblastoma Cell Lines (PDCL-GBM)

To this point, we have confirmed that concurrent introduction of large numbers of DSBs by C2K results in G2/M arrest followed by apoptosis thus laying the base for the possible use of C2K in SGT. To characterize the actual potential of C2K in killing tumor cells, we next compared its capability to trigger PCD with that of irradiation, a common treatment option in oncology. To do so, we subjected the U87 glioblastoma cell line to either C2K, irradiation, or a combination thereof and assessed the impact of the different treatments using a well-established colony-formation assay. We used low-level C2K transduction (20%) to study a realistic treatment scenario. Remarkably, despite this low transduction rate C2K more efficiently inhibited the formation and growth of colonies than the application of 2-Gy irradiation in both cell lines. Moreover, the combined application of C2K and irradiation had an additive effect (Figure S3). Interestingly, the U87 GBM cell line was described as markedly radio-resistant in comparison with other cancer cell lines [30].

These results prompted us to test whether the C2K approach efficiently triggers PCD in patient-derived cell-line (PDCL) models of glioblastoma (GBM). We chose GBM, as it is one of the most aggressive and difficult-to-treat cancers with a dismal prognosis. As compared to conventional cell lines, PDCL-GBM [23], often referred to as “stem-like” GBM cell lines [21,22] were shown to more accurately represent the biology [31,32] and therapy-responsiveness of GBM [21,22,33]. In a first experiment, we directly compared the induction of DSBs by C2K and 5-Gy irradiation in NCH644 cells by assessing the formation of eGFP-positive foci as described above. We found that C2K induced a substantial increase in DSB numbers in those patient-derived GBM cells, whereas 5-Gy irradiation had a much lower impact, particularly after 96 h. Importantly, when applied together the two treatments showed additive effects (Figure S4).

**Figure 3:**
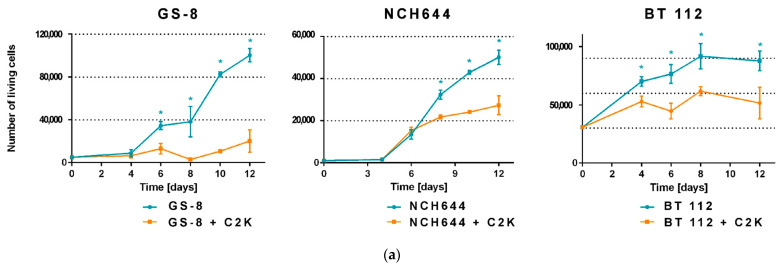

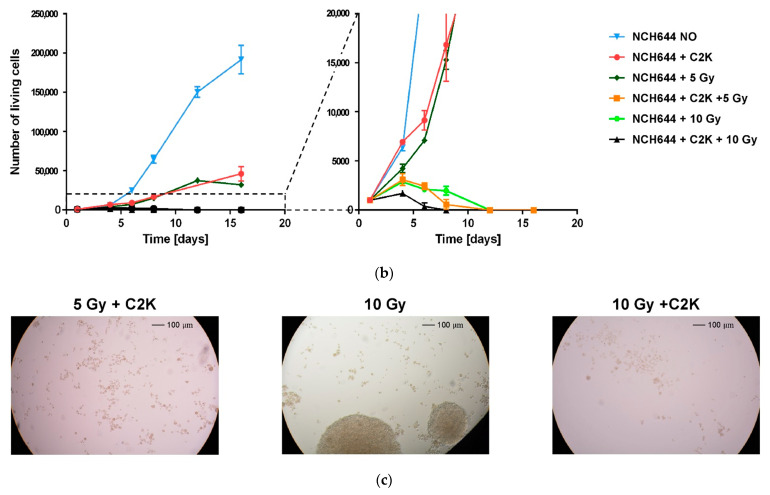
C2K efficiently inhibits growth, triggers PCD, and increases radiosensitivity in patient-derived glioblastoma cell lines (PDCL-GBM). (**a**) Inhibition of cell proliferation of three different PDCL-GBM by C2K. Data are presented as mean ± SEM, *n* = 3, * *p* < 0.05. (**b**) The efficiency of PCD induction in irradiation-resistant PDCL-GBM using C2K, high-dose irradiation, and combinations thereof. The number of living cells was assessed by MTT-assay. Data are represented as mean ± SEM, *n* = 3, * *p* < 0.05. (**c**) Photographs depict the largest spheres among all samples treated with 5 Gy + C2K, 10 Gy, and 10 Gy + C2K, respectively, 26 days after treatment. See also Figure S5.

In the next step, we used C2K on three different GMB-PDCLs (NCH644, BT112, GS-8) and found that it facilitated efficient growth inhibition in all of them (Figure 3a).

NCH644 cells were previously shown to be particularly resistant to radiotherapy. In fact, even after 10-Gy irradiation single NCH644 clones were found to restart growing [33]. Therefore, we asked whether a combination of C2K treatment (this time using high transduction rates) with irradiation might overcome this resistance (Figure 3b,c). As shown in Figure 3b, C2K as sole treatment triggered PCD as efficiently as 5-Gy irradiation reducing cell counts by approximately 75% at day 16. However, after both single treatments, cells were able to regrow (Figure 3b) and also formed spheres again (not shown). In contrast, combined application of C2K and 5-Gy irradiation resulted, similar to 10-Gy irradiation, in evidently complete suppression of NCH644 growth as measured with the MTT assay 16 days after irradiation (Figure 3b). In view of the earlier reported recovery of single NCH644 cells even after 10-Gy irradiation [33], we left the apparently dead cultures in the incubator. Strikingly, after app. 20 days we observed regrowth of single GBM spheres in two out of three samples irradiated with 10 Gy, whereas no sphere formation was seen after combination treatment 26 days post-treatment (Figure 3c and Figure S5). The higher efficacy of combined C2K + 5-Gy treatment indicates at least additive action and supports the potential of C2K as a sensitizing treatment for radio-resistant tumor cells.

## 4. Discussion

CRISPR-Cas9 originally represents an adaptive immune system in prokaryotes to protect the latter from invading DNA. We adapted this protective mechanism to be used as a suicide approach against cancer cells, i.e., not to edit a cell’s genome, but to destroy it. Given its original function, the introduction of our CRISPR-to-kill approach might be viewed as a “back-to-the-roots” for CRISPR/Cas.

Our work has established proof-of-concept as well as the main mechanisms of C2K-induced cell deaths. We have shown that concurrent introduction of large numbers of DSBs results in G2/M arrest followed by apoptosis.

An obvious limitation of the C2K approach as presented in this conceptual study is the lack of tumor specificity, which is, however, a common shortcoming of suicide systems. In fact, numerous approaches addressing this challenge have been developed and could be combined to ensure cancer-tissue specificity at different levels [34,35], e.g., delivery using targeted vectors [36,37], transcriptional and posttranscriptional targeting, e.g., using cell-specific promoters and/or miRNA target sequences [37,38,39], and/or application of switchable variants of Cas9 [40] and gRNA [41].

Importantly, induction of multiple DSBs by C2K can be expected to trigger PCD even in treatment-resistant as well as dormant (non-proliferative) cancer stem cells suggested to drive relapses after therapy [7,31], which addresses the main limitation of other suicide genes. Notably, C2K does not depend on the application of additional prodrugs, which might be associated with specific side effects (e.g., myelotoxicity of ganciclovir [42]). The efficacy of classical prodrug-based suicide systems is further limited by inefficient penetration of the prodrug into tumor tissue after systemic application, in particular in the case of brain tumors and the blood–brain barrier. Finally, the pronounced immunogenicity of Cas9 [43,44] can be predicted to cause a local inflammatory reaction thus ideally inducing a systemic anti-tumor immune response also directed against non-transduced cells. Similar to the concept of oncolytic viruses, such a systemic immune answer might be supported by the co-expression of immune-modulating factors.

Notably, we showed that C2K is easily designable to be species-specific. This property of C2K has the potential to be used in fighting parasitic diseases of humans or domestic animals. When adopted to certain bacterial genetic repeats, it could serve as a species-selective antibiotic.

## 5. Conclusions

In conclusion, we have provided proof of principle for a novel application of the CRISPR/Cas system as a suicide device for cancer gene therapy. We propose our C2K system as a promising new gene-therapy approach that warrants further in-depth investigation.

## Data Availability

Raw data can be made available upon reasonable request.

## Supplementary Materials

The following are available online at https://www.mdpi.com/article/10.3390/cancers13246306/s1, Figure S1: Alu-SINE sequence and vector design, Figure S2: Alu-C2K inhibits growth in human cells, Figure S3: Additive effect of C2K and irradiation on U87 glioma cells growth inhibition, Figure S4: C2K application results in higher DSBs number than high dose of irradiation, Figure S5: Additive effect of C2K and irradiation treatment on the NCH644 glioma cells growth inhibition, Table S1: Oligonucleotides used in the study.
