# Supplementary material for: CRISPR-to-Kill (C2K)–Employing the Bacterial Immune System to Kill Cancer Cells
Source: Cancers (Basel). 2021 Dec 15;13(24):6306. doi: 10.3390/cancers13246306 (PMC8699370; doi:10.3390/cancers13246306)
Supplement: Supplementary file 1 [file cancers-13-06306-s001.zip › cancers-1461331-supplementary.pdf]

cancers

MDPI

Supplementary Materials

CRISPR-to-Kill (C2K)–Employing the Bacterial Immune System to Kill Cancer Cells

Dawid Głów [1], Cecile L. Maire [2], Lea Isabell Schwarze [1], Katrin Lamszus [2] and Boris Fehse [1,*]

[1] Research Department, Cell and Gene Therapy, Department of Stem Cell Transplantation, University Medical Centre Hamburg-Eppendorf (UKE), 20246 Hamburg, Germany; d.glow@uke.de (D.G.); l.schwarze@uke.de (L.I.S.)
[2] Department of Neurosurgery, University Medical Center Hamburg-Eppendorf (UKE), 20246 Hamburg, Germany; cmaire@uke.de (C.L.M.); lamszus@uke.uni-hamburg.de (K.L.)
* Correspondence: fehse@uke.de; Tel.: +49-40-7410-55518; Fax: +49-40-7410-55468

A

Alu SINE

GGCCGGGCGCGGTGGCTCACGCCTGTAATCCCAGCACTTTGGGAGGCCGAGGCGGGCGGATCACG
AGGTCAGGAGATCGAGACCATCCTGGCCAACACGGTGAAACCCCGTCTCTACTAAAAATACAAAA
ATTAGCCGGGCGTGGTGGCGGGCGCCTGTAGTCCCAGCTACTCGGGAGGCTGAGGCAGGAGAATG
GCGTGAACCCGGGAGGCGGAGCTTGCAGTGAGCCGAGATCGCGCCACTGCACTCCAGCCTGGGCG
ACAGAGCGAGACTCCGTCTCAAAAAAAAAA

B

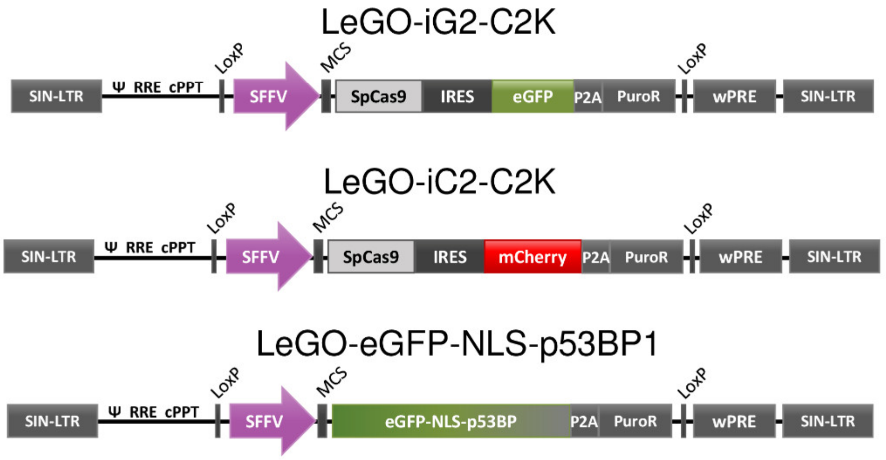

Figure S1. Target sequence in Alu SINE and design of used LeGO vectors. (**A**) Sequence of Alu SINE targeted with target sequence (Alu-gRNA used in the experiments is marked in orange, the PAM sequence in blue). (**B**) Graphical representation of the LeGO-iG2-C2K, LeGO-iC2-C2K, and LeGO-eGFP-NLS-p53BP vectors (not to scale).

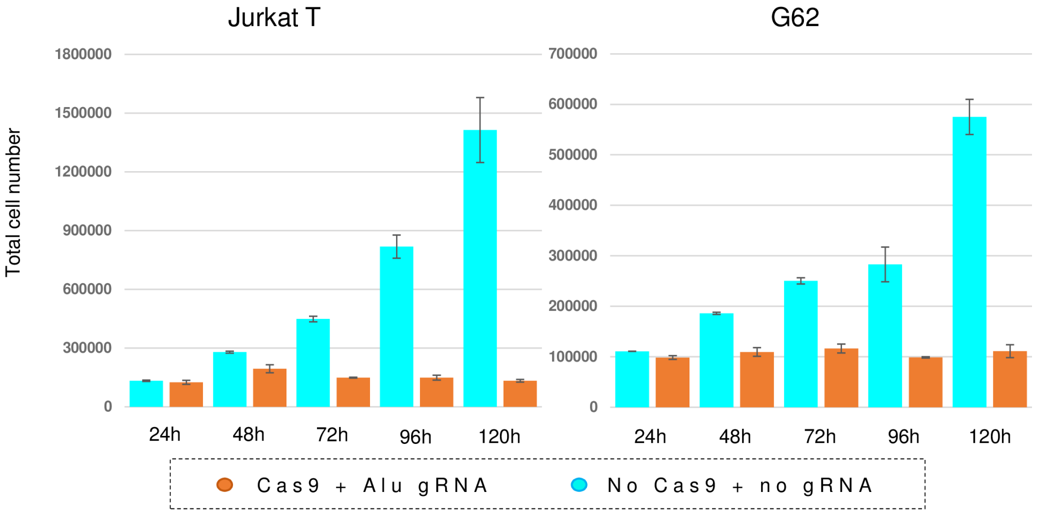

Figure S2. C2K-mediated growth suppression in human Jurkat and G62 cell lines. Growth inhibition of Jurkat T and G62 glioma cells after Alu-targeted C2K. Absolute numbers of living cells were counted by flow cytometry in defined volumes 24, 48, 72, 96 and 120h after transduction. Shown are cell numbers extrapolated to the volume of single wells. Data are presented as mean ± SEM, *n*=3.

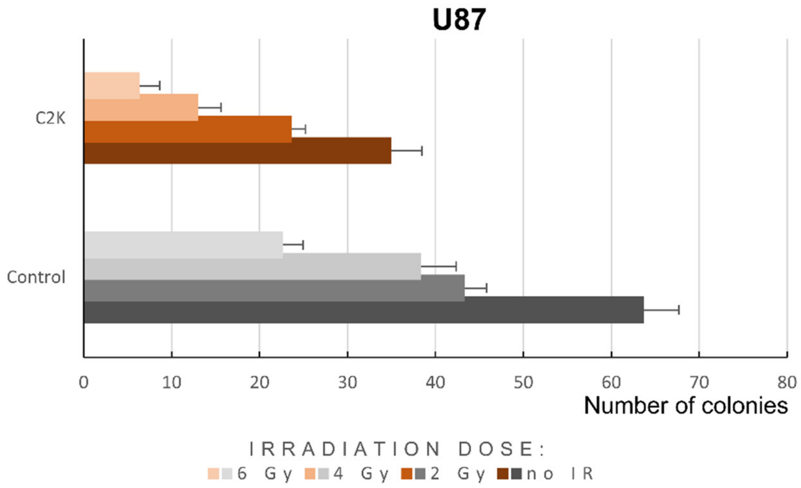

Figure S3. Synergistic effects of C2K and irradiation on U87 glioma cell growth inhibition. Growth inhibition of U87 glioma cells after Alu-targeted C2K and/or irradiation (2, 4, and 6 Gy) application measured by colony-formation assay. Data is presented as mean ± SEM, *n*=3.

NCH 644

	C2K	Irradiation dose	Number of analysed cells	Median number of foci
Day 1	-	-	453	18
	-	5 Gy	616	20
	+	-	600	24
	+	5 Gy	636	26
Day 4	-	-	1919	19
	-	5 Gy	1402	25
	+	-	954	38
	+	5 Gy	402	43

Number of foci: ≤20; ≤30; ≤40; >40

Figure S4. C2K is more efficient in inducing DSBs than high-dose irradiation. Induction of DNA DSBs (visualized as eGFP foci) in patient-derived NCH 644 glioma cells treated with C2K, 5 Gy, or combinations thereof. DSB induction was measured 1 and 4 days after treatment. C2K and irradiation show additive effects.

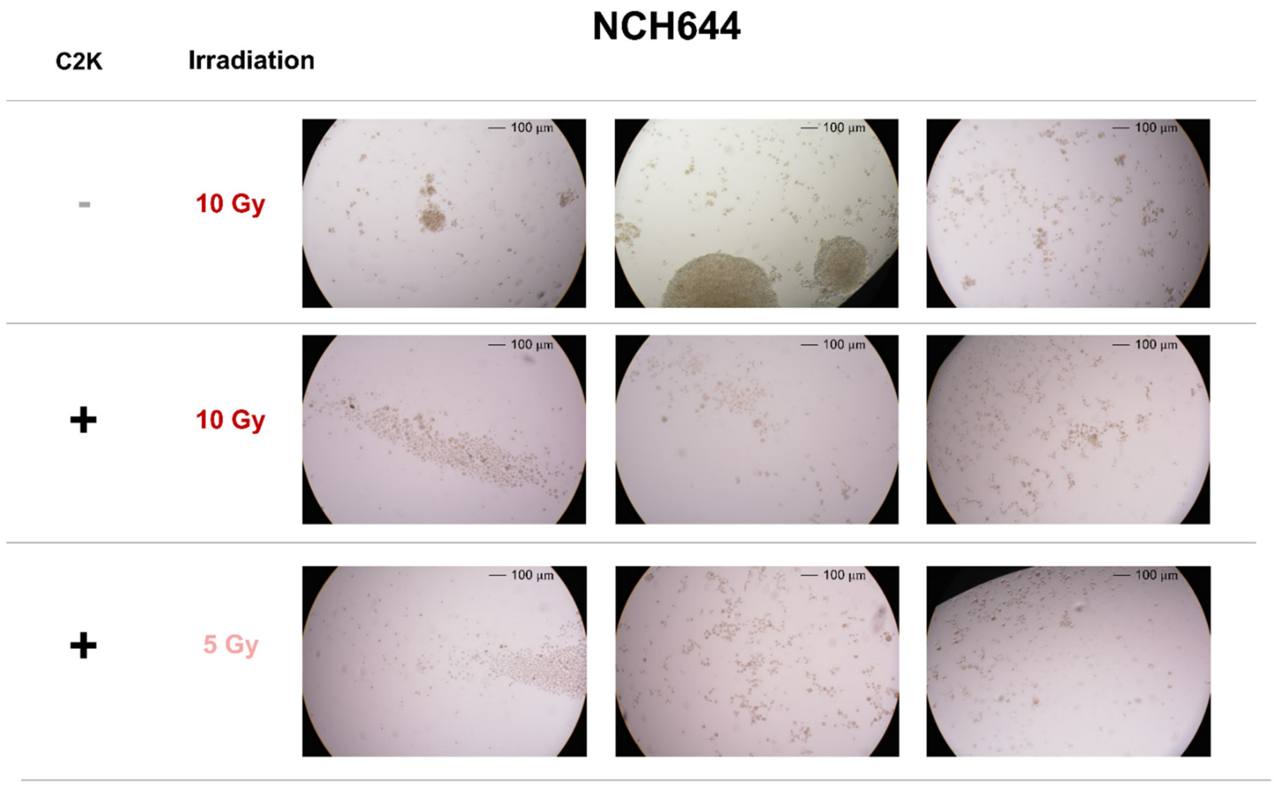

Figure S5. Outgrowth of resistant NCH 644 tumor cell spheres after 10-Gy irradiation. Regrowth of NCH 644 glioma cells was observed in 2 of 3 cultures 26 day after treatment by 10-Gy irradiation, but not after combined treatment with 5 Gy + C2K or 10 Gy + C2K.

Table S1. Oligonucleotides used in the study.

PRIMER NAME	SEQUENCE	REMARKS
F-Alu-gRNA	ACCGCGGTGCTGACCAGAAG	Contain Alu SINE targeting proto-spacer sequence
R-Alu-gRNA	AACGTTGTCGATGGAGTCGTCC	
nls53BP1 fw	GCGCGATCACATGGTCCTGCTG	Used to amplify nls53BP1 from pcDNA-FRT/T0-eGFPnls-53BP1. PCR product was cut with BsrGI + XbaI and cloned into LeGO-G2 simultaneously with the 2Apuro fragment.
nls53BP1 rv	TGCC**TCTAGA**ACTGCGCCGTTTCCGCTT	
2Apuro fw	CAGTTCTAGAGGCAGCGGCGCCACCAA	Used to amplify 2Apuro from LeGO-iC2puro+. PCR product was cut with XbaI x BsrGI and cloned into LeGO-G2 simultaneously with the nls53BP1 fragment.
2Apuro rv	GGAGCAACATAGTTAAGAATACCAGTCAATCTTTC	
